# Dataset of Jordanian university students’ psychological health impacted by using e-learning tools during COVID-19

**DOI:** 10.1016/j.dib.2020.106104

**Published:** 2020-07-31

**Authors:** Ahmad S Haider, Saleh Al-Salman

**Affiliations:** Applied Science Private University, Jordan

**Keywords:** Psychosomatic, COVID19, College students, Disorders, Stress, Tension, Emotional well-being, Jordan

## Abstract

A dataset was compiled to examine the psychosomatic impact of COVID-19′s e-learning digital tools on Jordanian university students’ well-being. In response to the state of emergency imposed by COVID-19, Jordanian universities switched to the online learning model as an alternative to traditional face-to-face education. The researchers designed a questionnaire that consists of two main sections; the first section included demographic information including gender, level/year, age, and cumulative average (GPA). The second section comprised five main constructs: (1) use of digital tools (mobile phone, laptop, i-pad) before and after COVID-19, (2) sleeping habits before and after COVID-19, (3) social interaction, (4) psychological state, and (5) academic performance. The researchers contacted different instructors teaching compulsory courses at four public and private universities and asked them to distribute the electronic questionnaire. Using the snowball sampling method, the questionnaire was delivered to students studying at the selected universities, and a total of 775 responses were received. The data were analyzed according to Likert's five-point scale, where frequencies and percentages were calculated. The data will be useful for researchers interested in studying the relationship between the e-learning model and psychosomatic disorders. Policymakers can use the data to identify university students’ emotional and psychological needs and propose practical solutions for their educational well-being.

**Specifications Table**

**Table:** 

**Subject**	Education, Psychology
**Specific subject area**	e-learning tools; psychosomatic disorders; university students
**Type of data**	Table Excel file
**How data were acquired**	Data were gathered using an online questionnaire that is launched on an online survey platform (Microsoft Forms) and then converted into .xlsx format. A Likert-type questionnaire was designed and administered in Arabic being the official language in Jordan. The questionnaire can be accessed online via the following links: **Arabic Link**: https://forms.office.com/Pages/ResponsePage.aspx?id=Huu9piR3ZUG3lmQANPUHuk7kvNATRIRNtS7aI8GqQPRURTNYV0pBMlA1OEhOQ1ZPQVEzR0pFVE81RS4u **English Translation**: https://forms.office.com/Pages/ResponsePage.aspx?id=Huu9piR3ZUG3lmQANPUHuoWAQDBppUdOmEPJ0Iicmj1URDBNM1FPVTJGQ1RRUk5FTTA5UDUwRkpPOS4u
**Data format**	Raw Analyzed
**Parameters for data collection**	The target population of the questionnaire was students of two public universities and two private universities in Jordan. The data were collected after the end of the second term of the academic year 2019/2020. The students were using the online learning platforms during this semester due to the novel coronavirus pandemic.
**Description of data collection**	An online questionnaire was given to different instructors teaching compulsory courses at four Jordanian public and private universities, and they were asked to distribute the questionnaire's link to their students. The snowball sampling technique helped in having more respondents as the students were advised to forward the link to colleagues studying at the same university.
**Data source location**	Institution: The University of Jordan, The Hashemite University, Applied Science Private University, and University of Petra City: Amman Country: Jordan 30.5852° N, 36.2384° E
**Data**	Dataset is uploaded on Mendeley
**Accessibility**	Repository name: Mendeley repository Direct URL to data: https://data.mendeley.com/datasets/thnzm3yk23/draft?a=f7af7bf4-8573-4141-a3a7-fe2884ba507e

## Value of the data

• The dataset is important because it contributes to identifying the university students’ psychosomatic disorders associated with emergency online learning.
• The dataset will be useful for researchers who want to examine the impact of quarantine, closures, social distancing, and isolation on the college students’ mental health and psychological well-being.
• The dataset can be used/reused for comparison purposes. For example, comparing it with other datasets collected from other countries, or with other datasets examining the impact of utilising both online and face-to-face-education (blended learning) on the students’ psychological state.
• The data will provide insights and guidance on how to improve the quality of learning and students’ mental health impacted by using digital learning tools based on the current emergency learning situation.
• The dataset will be useful to educational leaders and policymakers by helping them identify university students’ emotional and psychological needs and propose practical solutions for their educational well-being.
• The dataset can influence some aspects in the educational process including the number of assignments, methods of teaching, learning, and assessment.

## Data description

COVID-19 has affected different sectors in society, and education is not an exception. Schools and higher education institutions were among the most affected. In Jordan, the government imposed an emergency state through taking strict measures to prevent further spread of the virus. These measures included closing borders, suspending schools and universities, halting flights, banning gatherings, quarantine, and others. Students’ shifting to the e-learning model led to the prolonged use of digital e-learning tools including smartphones, laptops, and i-pad tablets, which ultimately affected their mental health and their psychological well-being. This dataset focuses on the impact of the excessive use of COVID-19′s e-learning digital tools on university students’ psychological and mental health. The dataset further investigates whether there is a correlation between the students’ prolonged use of e-learning digital tools, imposed by the COVID-19 crisis, and the psychosomatic symptoms and disorders [1,2].

A Likert-type questionnaire was administered in Arabic, being the official language in Jordan (see supplementary file 1). A version of the questionnaire is also available in English (see supplementary file 2). This dataset contains two main sections; the first section is demographic information, and the second section reports on the psychosomatic impact of COVID-19′s e-learning digital tools on university students’ well-being. A total of 775 responses were received. The raw data can be found at Mendeley dataset via the following link https://data.mendeley.com/datasets/thnzm3yk23/draft?a=f7af7bf4-8573-4141-a3a7-fe2884ba507e. The data were analyzed according to Likert's five-point scale, where frequencies and percentages were calculated.

The demographic information part aimed to gather information about gender, level/year (first/freshman, second/ sophomore, third/junior, fourth/senior, or other), age (18–24, 25–30, or 30+), cumulative average/GPA (+90 / 3.5+, 80–89 / 3.0–3.49, 70–79 / 2.5–2.99, 60–69 / 2.0–2.49, Below 60 / Below 2.0 or other) (Table 1).

**Table 1:** Demographic information.

Variable	Categories	Frequencies	Percent
**Gender**	Female	616	79.5%
	Male	159	20.5%
**Level/Year**	First/Freshman	120	15.5%
	Second/ Sophomore	199	25.7%
	Third/Junior	198	25.5%
	Fourth/Senior	203	26.2%
	Other	55	7.1%
**Age**	18–24	697	89.9%
	25–30	52	6.7%
	30+	26	3.4%
**cumulative average (GPA)**	+90 / 3.5+	131	16.9%
	80–89 / 3.0–3.49	307	39.6%
	70–79 / 2.5–2.99	263	33.9%
	60–69 / 2.0–2.49	67	8.6%
	Below 60 / Below 2.0	3	0.4%
	Other	4	0.5%

The second section consisted of five main constructs. The first construct is concerned with the use of digital tools (mobile phone, laptop, i-pad) before and after COVID-19. This construct identifies the type of digital tools used, length of use before and after COVID-19, and whether their excessive use for academic purposes led to distraction (Table 2). The second construct seeks to compare the students’ sleeping habits before and after COVID-19, and if their frequent use of e-learning tools interfered with their bedtime and wake-up (Table 3).

**Table 2:** Use of digital tools (mobile phone, laptop, i-pad).

A	Use of digital tools (mobile phone, laptop, i-pad)						
	**Item**		**Laptop**	**Mobile phone**	**I pad/ Tablet**	**Personal Computer**	**Other**
1	Which of the following digital tools do you usually use?	Before COVID 19	24.1%	62.3%	3%	1.5%	9%
		After COVID 19	48.1%	45.5%	2.5%	3%	1.3%

	**Item**		**1–3**	**3–6**	**6–9**	**9–12**	**+12**

2	How much time do you spend using the digital tools in learning?	Before COVID 19	64.9%	22.7%	9%	2.3%	1%
		After COVID 19	16.3%	32.5%	27.7%	16.9%	6.6%

	**Item**		**Strongly Agree**	**Agree**	**Uncertain**	**Disagree**	**Strongly Disagree**

3	I always use digital tools (mobile, laptop, i-pad) in studying.	Before COVID 19	13.3%	39.7%	26.2%	16.8%	4%
		After COVID 19	59.6%	27.2%	6.7%	4.5%	1.9%
4	When I use the mobile phone, tablet or laptop in e-learning, I cannot concentrate and I am distracted.	Before COVID 19	22.3%	31.7%	26.1%	16.9%	3%
		After COVID 19	36%	26.3%	17.3%	15.9%	4.9%

**Table 3:** Students’ sleeping habits.

	Sleeping habits						
	Item		Strongly Agree	Agree	Uncertain	Disagree	Strongly Disagree
5	I have fixed hours for bedtime and wake-up.	Before COVID 19	37.5%	40.8%	10.8%	8.1%	2.7%
		After COVID 19	9.7%	17.4%	17%	35%	20.9%
6	Prolonged use of digital tools for learning (mobile, laptop, i-pad) affected my sleeping habits.	Before COVID 19	19.5%	24.9%	27.4%	24.8%	3.5%
		After COVID 19	53.7%	27%	10.7%	7%	1.7%
7	Continuous exposure to electronic screens in online learning is tiring and exhausting.	Before COVID 19	36%	32.6%	17.2%	12.6%	1.5%
		After COVID 19	70.2%	21.4%	4.3%	2.6%	1.5%

The third construct aims to elicit responses on the students’ social behaviour and the extent to which the closures, lockdowns, and curfews, in addition to their prolonged use of e-learning tools, have impacted their everyday life routines (Table 4).

**Table 4:** Students’ social interaction and distance learning.

C	Social interaction					
	**Item**	**Strongly Agree**	**Agree**	**Uncertain**	**Disagree**	**Strongly Disagree**
8	The distance learning system, caused by the COVID-19 epidemic, resulted in social distancing.	38.7%	37.5%	13.8%	7.5%	2.5%
9	Prolonged use of digital tools (mobile, laptop, i-pad) causes students’ isolation.	48.3%	36.4%	9.2%	4.8%	1.4%
10	University learning contributes to strengthening the social personality of students.	46.8%	28.4%	14.3%	8.3%	2.2%
11	Staying home for long periods of time leads to lethargy and laziness.	60.9%	24.6%	9.6%	3.6%	1.2%

The fourth construct focused on the psychological state of students after having undergone unfavourable conditions during the COVID-19 crisis. This was reflected in cases of stress, frustration, tension, and depression (Table 5). The last construct seeks to probe into the consequences of the above factors, i.e. social and psychological, on the students’ academic performance and achievements (Table 6).

**Table 5:** Students’ Psychological state and distance learning.

D	Psychological State					
	**Item**	**Strongly Agree**	**Agree**	**Uncertain**	**Disagree**	**Strongly Disagree**
12	Prolonged use of e-learning tools often leads to boredom, nervousness, and tension.	54.7%	33.9%	7.7%	3.5%	0.1%
13	The psychological element is a key factor in the success of the educational process.	68.6%	26.6%	3%	1.7%	0.1%
14	Some students cannot afford buying all necessary digital tools, which is embarrassing and frustrating.	69%	26.3%	3.7%	0.9%	0%
15	I don't recommend continuing with the online learning model because it is socially and psychologically unhealthy.	47.2%	25.5%	18.3%	8.5%	0.4%
16	Measures of lockdown, closures, and quarantine, brought by COVID-19 caused stress, frustration, and depression.	58.8%	27.7%	8.9%	4.4%	0.1%

**Table 6:** Students’ academic performance and distance learning.

E	Academic performance					
	**Item**	**Strongly Agree**	**Agree**	**Uncertain**	**Disagree**	**Strongly Disagree**
17	Use of digital learning tools is responsible for my low academic performance.	49.9%	31.6%	11.6%	4.9%	1.9%
18	The volume of assignments via e-learning led to confusion, frustration and poor performance.	55.5%	27.9%	9.5%	5.4%	1.7%
19	Face-to-face interaction contributes significantly to boosting students’ academic achievement.	59.6%	30.5%	6.8%	1.9%	1.2%
20	Taking quizzes and exams online from home was not comfortable and made me nervous.	41.3%	24%	15.1%	12.4%	7.2%

## Experimental design, materials, and methods

The researchers adopted a descriptive survey design through developing a questionnaire to elicit students’ responses on the impact of the prolonged use of e-learning digital tools on their psychological well-being. Before designing the questionnaire, the researchers examined different questionnaires constructed for similar purposes [see [3], [4], [5]]. The researchers also consulted a jury of three experts for their feedback. The jury's comments were implemented before administering the questionnaires. Also, a test-retest on 50 students who did not participate in this research was conducted.

The researchers used Microsoft Forms to build an electronic version of the questionnaire.

The questionnaire of the current dataset has two main parts, the first part aims to gather demographic data, while the other part aimed to collect data on (1) use of digital tools (mobile phone, laptop, i-pad) before and after COVID-19, (2) sleeping habits, (3) social interaction, (4) psychological state, and (5) academic performance.

The questionnaire can be found via: https://forms.office.com/Pages/ResponsePage.aspx?id=Huu9piR3ZUG3lmQANPUHuk7kvNATRIRNtS7aI8GqQPRURTNYV0pBMlA1OEhOQ1ZPQVEzR0pFVE81RS4u

The English translation of the questionnaire: https://forms.office.com/Pages/ResponsePage.aspx?id=Huu9piR3ZUG3lmQANPUHuoWAQDBppUdOmEPJ0Iicmj1URDBNM1FPVTJGQ1RRUk5FTTA5UDUwRkpPOS4u

The questionnaire consisted of 20 items. In items 1 and 2, students were asked to select the digital tool they use the most before and after COVID-19 (item 1) and how many hours they spend using these digital tools before and after the pandemic (item 2). In the remaining 18 items, students were asked to fill in a Likert-type questionnaire ranging from ‘strongly agree’ to ‘strongly disagree’. Items 1 through 7 compare the respondents’ habits before and after COVID-19. Items 8 through 20 aim to collect responses about the impact of e-learning and prolonged use of digital tool on the students’ social, psychological, and academic well-being.

Four Jordanian universities, two public and two private, were selected. The researchers approached different lecturers who teach university compulsory courses to distribute the questionnaire. University compulsory courses were chosen as all students from different majors take them.

## Ethics statement

“All procedures performed in this work were in accordance with the ethical standards of the institutional and/or national research committee and with the 1964 Helsinki declaration and its later amendments or comparable ethical standards. Informed consent was obtained from all individual participants included in the data collection process”.

## Declaration of Competing Interest

The authors declare that they have no known competing financial interests or personal relationships that could have appeared to influence the work reported in this paper.

## Appendix Supplementary materials

Supplementary file 1: Arabic version of the questionnaire Image, application 1

Supplementary file 2: English translation of the questionnaire Image, application 2
