# Supplementary material for: Dataset of Jordanian university students’ psychological health impacted by using e-learning tools during COVID-19
Source: Data Brief. 2020 Jul 31;32:106104. doi: 10.1016/j.dib.2020.106104 (PMC7392902; doi:10.1016/j.dib.2020.106104)
Supplement: Supplementary file 1 — Arabic version of the questionnaire [file mmc1.docx]

**استبانة**

**Dataset of Jordanian University Students’ Psychological Health Impacted by Using E-learning Tools during COVID-19**

**المعلومات الديموغرافية:**

**الجنس**: ذكر أنثى

**المستوى / السنة**: أولى ثانية ثالثة رابعة غير ذلك __

**العمر**: 18-24 _____ 25-30 _____+ 30 _____

**المعدل التراكمي**:

+90 / 3.5+

80-89 / 3.0-3.49

70-79 / 2.5-2.99

60-69 / 2.0-2.49

أقل من /2.0أقل من 60

عزيزي الطالب:

لقد تم تصميم هذه الاستبانة لاستخلاص استجابات الطلاب حول "تأثير استخدام الموبايل والآي باد واللابتوب لفترات طويلة على الحالة النفسية لطلاب الجامعة أثناء أزمة كورونا المستجد".

ويؤكد الباحثون (القائمون على إجراء هذه الدراسة) بأنه سيتم استخدام إجاباتك على فقرات الاستبيان لأغراض بحثية فقط.

يرجى الاجابة على الأسئلة التالية.

وشكرا،

| **A** | **استخدام أدوات وسائل التعلم الإلكتروني** | | | | | | |
| --- | --- | --- | --- | --- | --- | --- | --- |
| 1 | أي من الأدوات الإلكترونية التالية تستخدمها عادة في التعلم؟ | قبل كورونا | اللابتوب | الموبايل | الاي باد | الكمبيوتر الشخصي | غير ذلك |
|  |  | بعد كورونا | اللابتوب | الموبايل | الاي باد | الكمبيوتر الشخصي | غير ذلك |
| 2 | كم من الوقت (بالساعات) تقضي في استخدام الأدوات الإلكترونية للتعلم؟ | قبل كورونا | 1-3 | 3-6 | 6-9 | 9-12 | +12 |
|  |  | بعد كورونا | 1-3 | 3-6 | 6-9 | 9-12 | +12 |
| 3 | أعتمد دائماً على استخدام أدوات التعلم الإلكتروني (الموبايل، اللابتوب، والآي باد... ) في دراستي. | قبل كورونا | أوافق بشدة | أوافق | محايد | غير موافق | غير موافق بشدة |
|  |  | بعد كورونا | أوافق بشدة | أوافق | محايد | غير موافق | غير موافق بشدة |
| 4 | عندما أستخدم الموبايل، أو اللابتوب، أو الآي باد للتعلم عن بعد، فإنني لا استطيع التركيز. | قبل كورونا | أوافق بشدة | أوافق | محايد | غير موافق | غير موافق بشدة |
|  |  | بعد كورونا | أوافق بشدة | أوافق | محايد | غير موافق | غير موافق بشدة |
| **B** | **أنماط النوم** | | | | | | |
| 5 | لدي ساعات محددة لوقت النوم والاستيقاظ. | قبل كورونا | أوافق بشدة | أوافق | محايد | غير موافق | غير موافق بشدة |
|  |  | بعد كورونا | أوافق بشدة | أوافق | محايد | غير موافق | غير موافق بشدة |
| 6 | استخدامي لأدوات التعلم الإلكتروني (الموبايل، أو اللابتوب، أو الآي باد) لفترات طويلة تسببت في تغيير أنماط النوم لدي. | قبل كورونا | أوافق بشدة | أوافق | محايد | غير موافق | غير موافق بشدة |
|  |  | بعد كورونا | أوافق بشدة | أوافق | محايد | غير موافق | غير موافق بشدة |
| 7 | التعرض المتواصل للشاشات الإلكترونية في التعلم عن بعد متعب ومرهق. | قبل كورونا | أوافق بشدة | أوافق | محايد | غير موافق | غير موافق بشدة |
|  |  | بعد كورونا | أوافق بشدة | أوافق | محايد | غير موافق | غير موافق بشدة |
| **C** | **التفاعل الاجتماعي** | | | | | | |
| 8 | يتسبب نظام التعلم عن بعد نتيجة تفشي وباء الكورونا إلى التباعد الإجتماعي. | | أوافق بشدة | أوافق | محايد | غير موافق | غير موافق بشدة |
| 9 | يؤدي الاستخدام المطول للأدوات الإلكترونية (الموبايل، أو اللابتوب، أو الآي باد ... ) الى العزلة. | | أوافق بشدة | أوافق | محايد | غير موافق | غير موافق بشدة |
| 10 | يتسبب نظام التعلم عن بعد في انعدام التفاعل الصفي (وجها لوجه) وضعف الشخصية الاجتماعية للطلاب. | | أوافق بشدة | أوافق | محايد | غير موافق | غير موافق بشدة |
| 11 | يؤدي البقاء في المنزل لفترات طويلة الى الخمول والكسل. | | أوافق بشدة | أوافق | محايد | غير موافق | غير موافق بشدة |
| **D** | **الحالة النفسية** | | | | | | |
| 12 | غالبا ما يؤدي الاستخدام المطوَل لوسائل التعلم الإلكتروني إلى الملل والعصبية والتوتر. | | أوافق بشدة | أوافق | محايد | غير موافق | غير موافق بشدة |
| 13 | تعتبر العوامل النفسية عنصراً أساسيا في نجاح العملية التعليمية. | | أوافق بشدة | أوافق | محايد | غير موافق | غير موافق بشدة |
| 14 | بعض الطلبة لا يستطيعون شراء جميع الأجهزة اللازمة للتعليم الإلكتروني، مما يتسبب لهم في الإحراج والإحباط . | | أوافق بشدة | أوافق | محايد | غير موافق | غير موافق بشدة |
| 15 | لا أوصي بالاستمرار في نظام التعلم عن بعد لأنه يتعارض مع الصحة النفسية والإجتماعية. | | أوافق بشدة | أوافق | محايد | غير موافق | غير موافق بشدة |
| 16 | تتسبب إجراءات الإغلاق والحظروالحجر الصحي المصاحبة لفايروس كورونا في التوتر والإحباط والاكتئاب. | | أوافق بشدة | أوافق | محايد | غير موافق | غير موافق بشدة |
| **E** | **الأداء الأكاديمي** | | | | | | |
| 17 | أنظمة التعلم عن بعد ليست بنفس الكفاءة والفاعلية التي يوفرها التعليم التقليدي مما يؤدي الى التراجع الأكاديمي للطالب. | | أوافق بشدة | أوافق | محايد | غير موافق | غير موافق بشدة |
| 18 | لقد تسبب الكم الكبير من الواجبات في عملية التعليم الالكتروني إلى الارتباك والإحباط وتردي الأداء الأكاديمي لدي. | | أوافق بشدة | أوافق | محايد | غير موافق | غير موافق بشدة |
| 19 | يساهم التفاعل الصفي (وجهًا لوجه) بشكل كبير في تعزيز النواحي الأكاديمية لدى الطلبة. | | أوافق بشدة | أوافق | محايد | غير موافق | غير موافق بشدة |
| 20 | أداء الاختبارات وبالذات النهائية في المنزل لم يكن مريحا وجعلني أشعر بالتوتر. | | أوافق بشدة | أوافق | محايد | غير موافق | غير موافق بشدة |

**شكراً لتعاونك!**
