# Supplementary material for: Dataset of Jordanian university students’ psychological health impacted by using e-learning tools during COVID-19
Source: Data Brief. 2020 Jul 31;32:106104. doi: 10.1016/j.dib.2020.106104 (PMC7392902; doi:10.1016/j.dib.2020.106104)
Supplement: Supplementary file 2 — English translation of the questionnaire [file mmc2.docx]

**Questionnaire**

**Dataset of Jordanian University Students’ Psychological Health Impacted by Using E-learning Tools during COVID-19**

1. **Demographic Information:**

**Gender:** Male: ____ Female: ____

**Level/Year**: First/Freshman: _____ Second/ Sophomore: _____

Third/Junior: _____ Fourth/Senior: ______

Other _____

**Age**: 18-24 _____ 25-30 ______ 30+ _____

**Your cumulative average (GPA):**

+90 / 3.5+

80-89 / 3.0-3.49

70-79 / 2.5-2.99

60-69 / 2.0-2.49

Below 60 / Below 2.0

**Dear Student:**

This research tool is designed to elicit students’ responses about the “Effect of Prolonged Smartphones, i-pads, and Laptop Use on the Psychological State of University Students during the COVID-19 Crisis". The researchers assure you that your responses to the questionnaire will be used only for research purposes.

Please respond to the following questions as indicated against each:

**Thank you!**

| **A** | **Use of digital tools (mobile phone, laptop, i-pad)** | | | | | | |
| --- | --- | --- | --- | --- | --- | --- | --- |
| 1 | Which of the following digital tools do you usually use? | **Before COVID 19** | Laptop | Mobile phone | I pad/ Tablet | Personal Computer | Other |
|  |  | **After**  **COVID 19** | Laptop | Mobile phone | I pad/ Tablet | Personal Computer | Other |
| 2 | How much time do you spend using the digital tools in learning? | **Before COVID 19** | 1-3 | 3-6 | 6-9 | 9-12 | +12 |
|  |  | **After**  **COVID 19** | 1-3 | 3-6 | 6-9 | 9-12 | +12 |
| 3 | I always use digital tools (mobile, laptop, i-pad) in studying. | **Before COVID 19** | Strongly Agree | Agree | Uncertain | Disagree | Strongly Disagree |
|  |  | **After**  **COVID 19** | Strongly Agree | Agree | Uncertain | Disagree | Strongly Disagree |
| 4 | When I use the mobile phone, tablet or laptop in e-learning I cannot concentrate and I am distracted. | **Before COVID 19** | Strongly Agree | Agree | Uncertain | Disagree | Strongly Disagree |
|  |  | **After**  **COVID 19** | Strongly Agree | Agree | Uncertain | Disagree | Strongly Disagree |
| **B** | **Sleeping Habits** | | | | | | |
| 5 | I have fixed hours for bed time and wake up. | **Before COVID 19** | Strongly Agree | Agree | Uncertain | Disagree | Strongly Disagree |
|  |  | **After**  **COVID 19** | Strongly Agree | Agree | Uncertain | Disagree | Strongly Disagree |
| 6 | Prolonged use of digital tools for learning (mobile, laptop, i-pad) affected my sleeping habits. | **Before COVID 19** | Strongly Agree | Agree | Uncertain | Disagree | Strongly Disagree |
|  |  | **After**  **COVID 19** | Strongly Agree | Agree | Uncertain | Disagree | Strongly Disagree |
| 7 | Continuous exposure to electronic screens in online learning is tiring and exhausting. | **Before COVID 19** | Strongly Agree | Agree | Uncertain | Disagree | Strongly Disagree |
|  |  | **After**  **COVID 19** | Strongly Agree | Agree | Uncertain | Disagree | Strongly Disagree |
| **C** | **Social Interaction** | | | | | | |
| 8 | Prolonged use of digital tools (mobile, laptop, i-pad) results in social distancing. | | Strongly Agree | Agree | Uncertain | Disagree | Strongly Disagree |
| 9 | Prolonged use of digital tools (mobile, laptop, i-pad) causes students’ isolation. | | Strongly Agree | Agree | Uncertain | Disagree | Strongly Disagree |
| 10 | University learning contributes to strengthening the social personality of students. | | Strongly Agree | Agree | Uncertain | Disagree | Strongly Disagree |
| 11 | Staying home for long periods of time leads to lethargy and laziness. | | Strongly Agree | Agree | Uncertain | Disagree | Strongly Disagree |
| **D** | **Psychological State** | | | | | | |
| 12 | Prolonged use of e-learning tools often leads to boredom, nervousness, and tension. | | Strongly Agree | Agree | Uncertain | Disagree | Strongly Disagree |
| 13 | The psychological element is a key factor in the success of the educational process. | | Strongly Agree | Agree | Uncertain | Disagree | Strongly Disagree |
| 14 | Some students cannot afford buying all necessary digital tools, which is embarrassing and frustrating. | | Strongly Agree | Agree | Uncertain | Disagree | Strongly Disagree |
| 15 | I don’t recommend continuing with the online learning model because it is socially and psychologically unhealthy. | | Strongly Agree | Agree | Uncertain | Disagree | Strongly Disagree |
| 16 | Measures of lockdown, closures, and quarantine, brought by COVID-19 caused stress, frustration, and depression. | | Strongly Agree | Agree | Uncertain | Disagree | Strongly Disagree |
| **E** | **Academic Performance** | | | | | | |
| 17 | Use of digital learning tools is responsible for my low academic performance. | | Strongly Agree | Agree | Uncertain | Disagree | Strongly Disagree |
| 18 | The volume of assignments via e-learning led to confusion, frustration and poor performance. | | Strongly Agree | Agree | Uncertain | Disagree | Strongly Disagree |
| 19 | Face-to-face interaction contributes significantly to boosting students’ academic achievement. | | Strongly Agree | Agree | Uncertain | Disagree | Strongly Disagree |
| 20 | Taking quizzes and exams online from home was not comfortable and made me nervous. | | Strongly Agree | Agree | Uncertain | Disagree | Strongly Disagree |

**Thank You!**
